# Inhibition of neuroinflammation and neuronal damage by the selective non-steroidal ERβ agonist AC-186

**DOI:** 10.1007/s00011-024-01952-y

**Published:** 2024-10-03

**Authors:** Folashade O. Katola, Misturah Y. Adana, Olumayokun A. Olajide

**Affiliations:** 1 Department of Pharmacy, School of Applied Sciences, University of Huddersfield, Huddersfield, HD1 3DH UK; 2 Department of Anatomy, Faculty of Basic Medical Sciences, College of Health Sciences, University of Ilorin, Ilorin, Nigeria; 3 Current Address: Peter O’Donnell Brain Institute, UT Southwestern Medical Center, Dallas, TX 75390 USA

**Keywords:** AC-186, Anti-inflammatory, Neuroprotective, NF-κB, ERβ

## Abstract

**Background:**

AC-186 (4-[4-4-Difluoro-1-(2-fluorophenyl) cyclohexyl] phenol) is a neuroprotective non-steroidal selective oestrogen receptor modulator. This study investigated whether inhibition of neuroinflammation contributed to neuroprotective activity of this compound.

**Methods:**

BV-2 microglia were treated with AC-186 (0.65–5 μM) prior to stimulation with LPS (100 ng/mL). Levels of pro-inflammatory mediators and proteins were then evaluated.

**Results:**

Treatment of LPS-activated BV-2 microglia with AC-186 resulted in significant (*p* < 0.05) reduction in TNFα, IL-6, NO, PGE_2_, iNOS and COX-2. Further investigations showed that AC-186 decreased LPS-induced elevated levels of phospho-p65, phospho-IκBα and acetyl-p65 proteins, while blocking DNA binding and luciferase activity of NF-κB. AC-186 induced significant (*p* < 0.05) increase in protein expression of ERβ, while enhancing ERE luciferase activity in BV-2 cells. Effects of the compound on oestrogen signalling in the microglia was confirmed in knockdown experiments which revealed a loss of anti-inflammatory activity following transfection with ERβ siRNA. In vitro neuroprotective activity of AC-186 was demonstrated by inhibition of activated microglia-mediated damage to HT-22 neurons.

**Conclusions:**

This study established that AC-186 produces NF-κB-mediated anti-inflammatory activity, which is proposed as a contributory mechanism involved in its neuroprotective actions. It is suggested that the anti-inflammatory activity of this compound is linked to its agonist effect on ERβ.

**Supplementary Information:**

The online version contains supplementary material available at 10.1007/s00011-024-01952-y.

## Introduction

Neuroinflammation continues to be a major focus of investigations into the pathobiology and pharmacology of Alzheimer’s disease (AD) and other neurodegenerative disorders including Parkinson’s disease (PD) and multiple sclerosis (MS). In fact, research focusing on neuroinflammation as a modifiable mechanism in the pathobiology of AD has expanded, with genetic studies linking the condition to genes uniquely expressed by brain myeloid cells such as the microglia [1, 2]. The roles of microglia in AD have been substantiated in studies demonstrating involvement of the cells in AD pathology [3]. Furthermore, post-mortem analyses of brains of AD patients have shown changes which were consistent with chronic inflammation [4–6].

Investigations focusing on new AD drugs targeting neuroinflammation have been promising. NE3107, a brain permeable anti-inflammatory drug which targets NF-κB mediated release of pro-inflammatory mediators is currently being investigated in Phase III clinical trials [7, 8]. Furthermore, the human anti-IL-1β monoclonal antibody Canakinumab (ACZ885), is an anti-inflammatory intervention for AD which is currently undergoing Phase II clinical trial [9]. Consequently, anti-inflammatory strategies will continue to provide reliable platforms for new AD therapeutics.

**Fig. 1:**
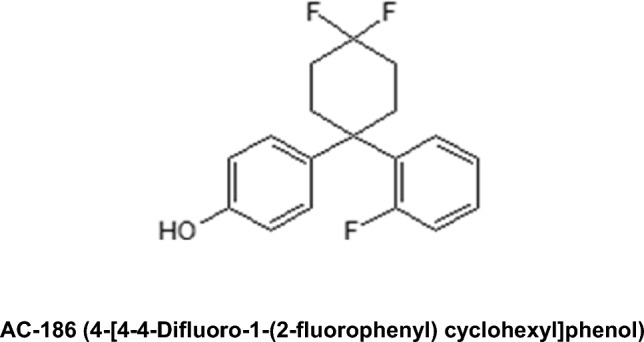
Chemical structure of AC-186

AC-186 (4-[4-4-Difluoro-1-(2-fluorophenyl) cyclohexyl] phenol) (Fig. 1) is a non_-_steroidal selective oestrogen receptor modulator (SERM), which has been previously investigated and shown to produce neuroprotective effects in animal models of PD, AD and MS [10–12]. This study was therefore designed to elucidate the involvement of neuroinflammatory signalling mechanisms in the neuroprotective activity of AC-186.

## Materials and methods

### Drugs and chemicals

AC-186 was purchased from Tocris, dissolved in dimethylsulfoxide (DMSO) to a concentration of 0.01 M and aliquots stored at − 20 °C. Lipopolysaccharide (LPS) from *Salmonella enterica* serotype typhimurium was purchased from Caltag Medsystems (UK). AC-186 was dissolved in DMSO (effective concentration of DMSO in culture medium at ≤ 0.2%), and investigated at concentrations of 0.625, 1.25, 2.5 and 5 µM.

### Cell culture

BV-2 microglia cell line (ICLCATL03001) was purchased from Interlab Cell Line Collection (Banca Biologica e Cell Factory, Italy). The cells were cultured in RPMI medium supplemented with 10% foetal bovine serum, 2 mM L-glutamine (Sigma), 100 mM sodium pyruvate (Sigma), 100 U/mL penicillin and 100 mg/mL streptomycin (Sigma). HT-22 neuronal cells were kindly gifted by Dr Jeff Davis (Swansea University, UK), maintained in DMEM supplemented with 10% FBS, 100 mM sodium pyruvate (Sigma), 100 U/mL penicillin and 100 mg/mL streptomycin (Sigma). All cell lines were cultured in a 5% CO_2_ incubator at 37 °C.

### Determination of BV-2 microglia viability

Cultured BV-2 microglia were treated with AC-186 for 30 min, followed by incubation with (100 ng/mL) for a further 24 h. Thereafter, medium was changed to 200 µL MTT (Sigma, UK) solution (0.5 mg/mL) and incubated in 5% CO_2_ incubator at 37 °C for 4 h. MTT solution was gently removed. Then, 150 µL DMSO was added to form purple-formazan crystals. Absorbance was read at 570 nm using Tecan F50 microplate reader.

### Production of pro-inflammatory mediators

Following treatment with AC-186 (0.625–5 μM), BV-2 microglia were stimulated with LPS (100 ng/mL) for 24 h. This was followed by collection of culture supernatants, which were then analysed for levels of pro-inflammatory cytokines (TNFα, and IL6) using mouse ELISA kits (Biolegend), according to the manufacturer’s instructions. Levels of nitrite in supernatants was determined as a measure of nitric oxide (NO) release using the Griess assay kit (Promega, UK). PGE_2_ levels were determined with a PGE_2_ enzyme immunoassay (EIA) kit (Arbor Assays, USA).

### Western blotting

Equal amounts of cell lysates (20–30μg) from treated BV-2 cells were subjected to SDS-PAGE under reducing conditions. This was followed by transfer to polyvinylidene fluoride (PVDF) membrane (Millipore), which was then blocked for 60 min at room temperature, washed with Tris-buffered saline + 0.1% Tween 20 (TBS-T) and incubated overnight at 4 °C with the following primary antibodies: rabbit anti-iNOS (Cell signalling, 1:1000), rabbit anti-COX-2 (Cell signalling, 1:1000), rabbit anti-phospho-p65 (Cell signalling, 1:1000), rabbit anti-phospho-IκBα (Abcam, 1:5000), rabbit anti-acetyl-p65 (Cell Signalling, 1:1000), rabbit anti-ERβ (Santa Cruz, 1:500), rabbit anti-SIRT-1 (Santa Cruz, 1:1000), rabbit anti-lamin B1 (Santa Cruz, 1:1000), and rabbit anti-actin (Sigma, 1:1000). Thereafter, membrane was incubated with Alexa Fluor 680 goat anti-rabbit secondary antibody (1:10,000; Thermo Scientific) at room temperature for 60 min. Blots were detected using LI-COR Odyssey Imager. All western blot experiments were carried out at least 3 times and quantified with image J software (National Institutes of Health).

### Transcription factor assays

Nuclear extracts from LPS-stimulated BV-2 cells pre-treated with AC-186 (0.625–5 μM) were used to evaluate DNA binding by NF-κB, using a transcription factor assay kit (Abcam). This assay utilised a 96-well plate with a double stranded DNA sequence containing the NF-κB response element (5’–GGGACTTTCC–3’) immobilised onto the bottom of the wells of a 96-well plate. Prior to the addition of nuclear extracts, a binding buffer was added to each well. The plate was then covered and rocked at 100 rpm for 60 min at room temperature. This was followed by addition of NF-κB antibody for 60 min, and then goat anti-rabbit HRP-conjugated secondary antibody for another 60 min. Absorbance was read at 450 nm in a Tecan F50 microplate reader.

### Transient transfection and luciferase reporter gene assays

Luciferase reporter gene assays were used to evaluate NF-κB-mediated gene expressions in BV-2 microglia. At 60% confluence, magnetofection (OZ Biosciences) was used to transfect BV-2 cells with a Cignal^®^ NF-κB luciferase reporter vector containing a firefly luciferase gene under the control of NF-κB responsive element [13, 14]. Similar procedures were used to transfect cells with a Cignal^®^ oestrogen receptor element (ERE) luciferase reporter vector. Transfection was followed by treatment with AC-186 (0.625–5 μM) and stimulation. NF-κB and ERE luciferase activities were evaluated with a Dual-Glo luciferase assay kit (Promega, UK). Luminescence was measured using POLARstar Optima microplate reader (BMG Labtech).

###  siRNA experiments

Knockdown of ERβ gene expression was achieved by changing BV-2 cell culture medium to Opti-MEM reduced-serum medium (Gibco™), followed by a 2-h incubation at 37 °C. Transfection reagent/siRNA complexes were prepared with 1.8 µl of Glial Mag (OZ Biosciences) and 2 µl of control siRNA (sc-37007; Santa Cruz Biotechnology) or ERβ siRNA (sc-35326; Santa Cruz Biotechnology) diluted in 200 µl Opti-MEM medium. The complexes were incubated at room temperature for 30 min and then added to the cells, followed by magnetofection. Twenty-four hours after magnetofection, Opti-MEM was replaced with serum-free RPMI followed by incubation at 37 °C for 2 h. This was followed by treatment with AC-186 (5 µM) and stimulation with LPS (100 ng/mL) to determine the impact of ERβ gene knockdown on anti-inflammatory activity of the compound. Culture supernatants were analysed for levels of TNFα and IL-6 ELISA.

### Transwell co-culture of BV-2 microglia and HT-22 neurons

Transwell co-culture experiments were used to determine effects of AC-186 treatment on neuroinflammation-mediated neurodegeneration, as described earlier [15]. BV-2 microglia were seeded out at a density of 5 × 10^4^ in transwell inserts (Corning; pore size 0.4 μm) and placed above HT-22 neurons in a 6-well plate. Once co-culture is established, AC-186 (5 μM) was added to BV-2 cells, followed by stimulation with (1 μg/mL) for 48 h. Thereafter, viability of adjacent HT-22 neurons was evaluated with the CyQUANT™ LDH cytotoxicity assay (Invitrogen), according to the manufacturer’s instructions. Culture supernatants were collected and analysed for levels of TNFα and IL-6 using mouse ELISA kits (Biolegend). In separate experiments, viability of neurons was assessed using the MTT assay.

### Statistical analysis

Data are presented as mean ± SEM of at least 3 independent experiments and analysed with one-way analysis of variance (ANOVA) followed by a post hoc Tukey multiple comparisons test. Data from siRNA experiments were analysed with two-way ANOVA followed by post hoc Tukey test. Statistical analyses were carried out with GraphPad Prism Software version 10.0.

## Results

### AC-186 did not affect the viability of LPS-stimulated BV-2 microglia

**Fig. 2:**
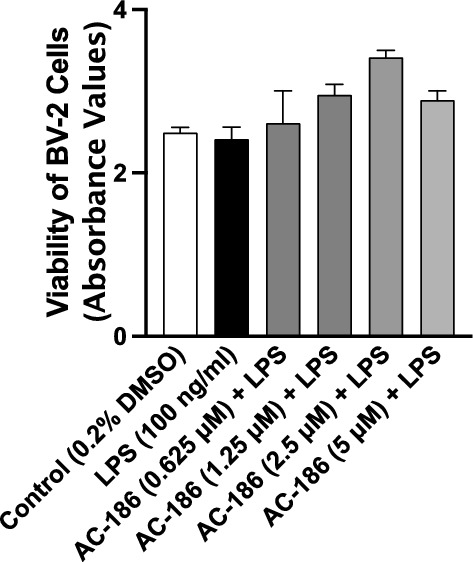
AC-186 (0.625, 1.25, 2.5 and 5 µM) did not reduce viability of BV-2 microglia stimulated with LPS (100 ng/mL)

BV-2 microglia were treated with AC-186 for 30 min, followed by stimulation with LPS (100 ng/mL) for a further 24 h. Results of MTT assay showed that at concentrations 0.625 µM, 1.25 µM, 2.5 µM and 5 µM, the compound did not reduce cell viability, when compared with cells incubated with 0.2% DMSO (Fig. 2).

### AC-186 decreased levels of TNFα and IL-6 in LPS-stimulated BV-2 microglia

**Fig. 3:**
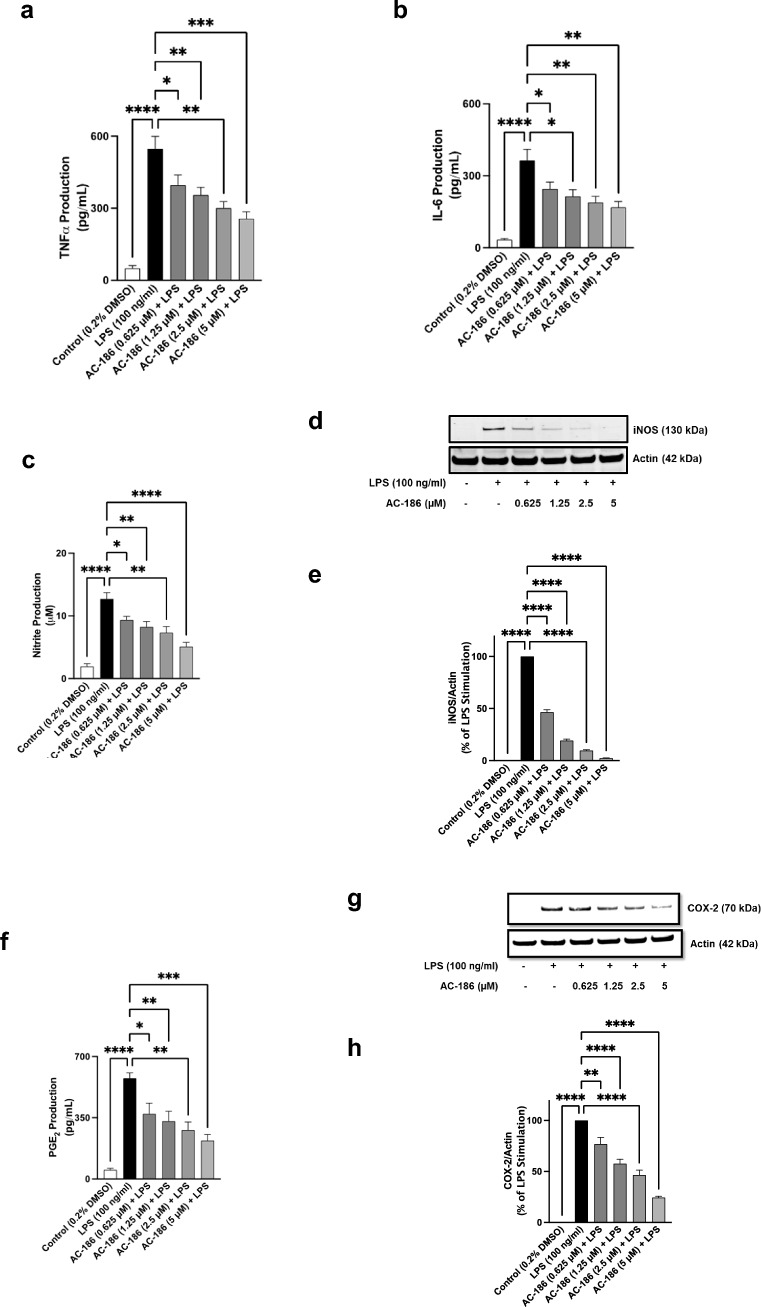
AC-186 suppressed TNFα and IL-6 production in BV-2 microglia cells. Microglia cells were treated with LPS (100 ng/mL) in the absence or presence of indicated concentrations of AC-186 for 24 h. TNFα (3A) and IL-6 (3B)  levels were measured using ELISA. Data are expressed as the mean ± SEM (n = 3). **p < 0.01, ***p < 0.001, ****p < 0.0001 versus LPS stimulation alone. AC-186 suppressed NO production and iNOS protein expression in BV-2 microglia cells. Microglia cells were treated with LPS (100 ng/mL) in the absence or presence of indicated concentrations of AC-186 for 24 h. Nitrite levels were measured using the Griess reaction (3C). BV-2 microglia cell lysates were subjected to Western blot for iNOS (3D). Levels of iNOS were normalised actin levels and expressed as a relative change in comparison with the LPS stimulation, which was set at 100% (3E). Data are expressed as the mean ± SEM (n = 3). *p < 0.05, ***p < 0.001, ****p < 0.0001 versus LPS stimulation alone. AC-186 suppressed PGE_2_ production and COX-2 protein expression in BV-2 microglia cells. Microglia cells were treated with LPS (100 ng/mL) in the absence or presence of indicated concentrations of AC-186 for 24 h. PGE_2_ levels were measured using enzyme immunoassay (3F). BV-2 microglia cell lysates were subjected to Western blot for COX-2 (3G). Levels of COX-2 were normalised actin levels and expressed as a relative change in comparison with the LPS stimulation, which was set at 100% (3H). Data are expressed as the mean ± SEM (n = 3). **p < 0.01, ***p < 0.001, ****p < 0.0001 versus LPS stimulation alone

Stimulation of BV-2 cells with LPS (100 ng/mL) for 24 h resulted in significant (*p* < 0.0001) increase in the release of TNFα, when compared to unstimulated BV-2 cells (Fig. 3a). However, pre-treatment of cells with 0.625 µM, 1.25 µM, 2.5 µM and 5 µM of AC-186 prior to LPS stimulation resulted in significant (*p* < 0.05) reduction in TNFα production, when compared to stimulation of cells with LPS alone (Fig. 3a). Similarly, incubation of BV-2 microglia with LPS for 24 h resulted in an elevated production of IL-6 in comparison to unstimulated cells. This increase in IL-6 production was however significantly reduced (*p* < 0.05) when cells were treated with AC-186 (0.625 µM, 1.25 µM, 2.5 µM and 5 µM) prior to activation with LPS (Fig. 3b).

### AC-186 reduced LPS-induced NO production/iNOS protein expression

Nitrite production in cells is taken as a surrogate marker for nitric oxide production. Analyses of culture supernatants revealed that stimulation of BV-2 microglia with LPS resulted in significant (*p* < 0.0001) increase in the production of nitrite, in comparison with unstimulated cells. In the presence of 0.625 µM, 1.25 µM, 2.5 µM and 5 µM of AC-186, nitrite concentration was reduced from ~ 12.7 μM to ~ 9.4 μM, ~ 8.2 μM, ~ 7.3 μM and ~ 5.1 μM, respectively (Fig. 3c).

Immunoblotting of cell lysates further showed that pre-treatment with AC-186 (0.625 µM, 1.25 µM, 2.5 µM and 5 µM) resulted in reduction of iNOS protein expression to 46.5%, 19.3%, 9.7% and 2.4%, respectively when compared to LPS stimulation (100%) (Fig. 3d and e).

### AC-186 reduced PGE_2_ release/COX-2 protein expression

The outcome of enzyme immunoassays on culture supernatants showed that stimulation of BV-2 microglia with LPS (100 ng/mL) resulted in elevated (*p* < 0.001) production of PGE_2_, when compared to unstimulated cells (Fig. 3f). However, pre-treating cells with AC-186 (0.625–5 µM) prior to LPS resulted in significant (*p* < 0.05) reduction in increased secretion of PGE_2_, in comparison with LPS stimulation alone.

Results of immunoblotting in Fig. 3g and h show that significant increase in COX-2 protein expression induced by LPS was reduced in a -concentration dependent fashion by AC-186 (0.625–5 µM).

### AC-186 targets NF-κB  signalling to produce anti-inflammatory activity

Based on results showing that AC-186 could reduce the production of pro-inflammatory mediators in LPS-activated BV-2 cells, experiments were conducted to evaluate the effects of the compound on mechanisms and molecular targets involved in the activation of the NF-κB transcription factor.

**Fig. 4:**
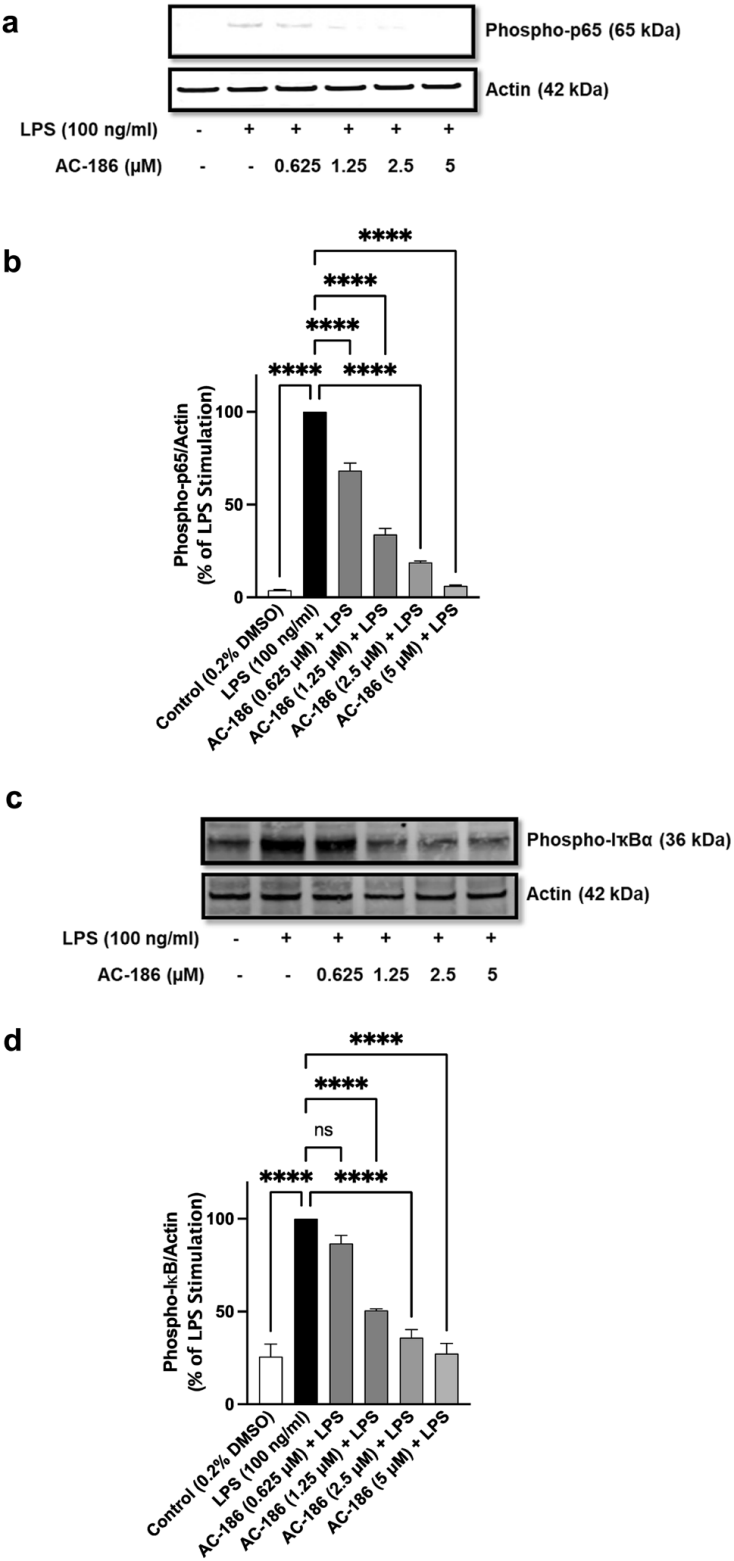

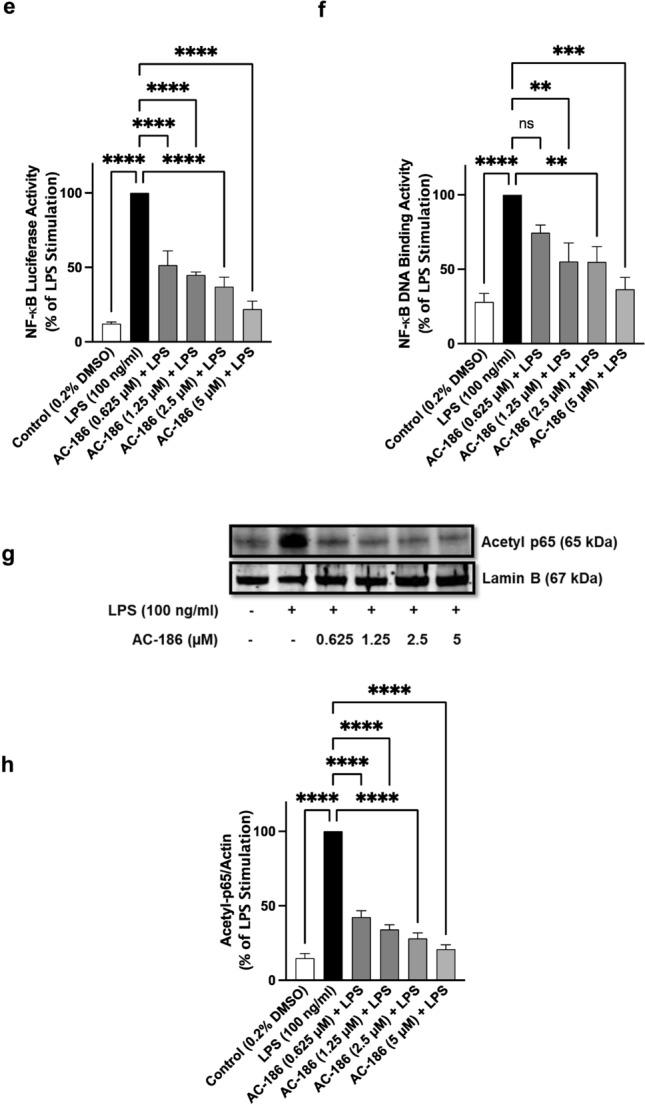
Inhibition of LPS-induced NF-κB activation by AC-186 in BV-2 cells. Cells were activated with LPS (100 ng/mL) with or without AC-186 (0.625–5 µM). Lysates of treated cells were used to detect phospho-p65 (4A, 4B) and phospho-IκBα (4C, 4D) expression. Actin was used as an internal loading control. Data are expressed as the mean ± SEM (n = 3). ns-not significant at *p* < 0.05, ****p < 0.0001 versus LPS stimulation alone. (4E). AC-186 inhibited LPS-induced NF-κB-dependent gene expression in BV-2 cells. Transfected cells were treated with AC186 (0.625–5 µM) and stimulated with LPS (100 ng/mL) for 6 h, followed by luciferase reporter gene assay. (4F) Reduction in LPS-induced increased binding of p65 NF-κB to consensus sites by AC-186. BV-2 cells were treated with AC186 (0.625–5 µM) and stimulated with LPS (100 ng/mL) followed by NF-κB transcription factor assay on nuclear extracts. Data are expressed as the mean ± SEM (n = 3). ns-not significant at *p* < 0.05, **p < 0.01, ***p < 0.001, ****p < 0.0001 versus LPS stimulation alone. (4G and 4H) AC-186 interferes with acetylation of p65 sub-unit following activation with LPS. AC-186-treated BV-2 microglia were stimulated with LPS (100 ng/mL). Western blotting on nuclear extracts showed reduction in protein expression of acetyl-p65. Lamin B was used as an internal loading control. Data are expressed as the mean ± SEM (n = 3). ****p < 0.0001 versus LPS stimulation alone

One of the upstream mechanisms involved in the activation of NF-κB is the phosphorylation of the p65 subunit and IκB complex in the cytoplasm. Immunoblotting analyses of BV-2 microglia stimulated with LPS (100 ng/mL) showed a significant (*p* < 0.001) increase in protein expression of the phospho-p65 subunit (Fig. 4a and b). When cells were treated with AC-186 (0.625–5 µM) prior to LPS stimulation, a concentration-dependent and significant (*p* < 0.001) reduction in phospho-p65 protein expression was observed. Furthermore, in the presence 1.25 µM, 2.5 µM, and 5 µM of AC-186, there was a concentration-dependent and significant (*p* < 0.0001) reduction in LPS-induced increased expression of phospho-IκBα protein (Fig. 4c and d). Interestingly significant (*p* < 0.05) reduction in LPS-induced increased phospho-IκBα protein levels was not observed with the lowest concentration of the compound (0.625 µM) investigated (Fig. 4c and d).

LPS-mediated phosphorylation of p65 subunit and IκBα result in the degradation of the latter and translocation of the former into the nucleus where it regulates the expression of pro-inflammatory genes including iNOS, COX-2 and the pro-inflammatory cytokines. Based on results showing inhibitory effects of AC-186 on the production of pro-inflammatory mediators, as well as phosphorylation of p65 in the cytoplasm, its effect on nuclear transactivation of NF-κB p65 was investigated.

Results of these experiments revealed that stimulation of BV-2 microglia with LPS (100 ng/mL) significantly enhanced (*p* < 0.001) NF-κB luciferase activity, and thus its transcriptional activity when compared with unstimulated cells (Fig. 4e). LPS-induced increased NF-κB luciferase activity was however reduced from 100% to 51.6%, 44.9%, 37% and 22% by 0.625, 1.25, 2.5 and 5 µM concentrations of AC-186, respectively (Fig. 4e).

NF-κB regulates gene transcription by binding to specific kappa B (κB) sites in the DNA. Results showing that AC-186 could reduce NF-κB transcriptional activity prompted investigations to determine whether the compound could interfere with ability of NF-κB to bind to DNA consensus sites. ELISA-based DNA binding assays revealed that the binding activity of nuclear NF-κB-p65 was significantly (*p* < 0.001) increased following stimulation of BV-2 cells with LPS (100 ng/mL) (Fig. 4f). These experiments also revealed that pre-treatment with AC-186 (1.25, 2.5 and 5 µM) prior to LPS stimulation resulted in decreased NF-κB-p65 DNA binding activity, whereas at 0.625 µM the compound did not produce significant (*p* < 0.05) reduction in DNA binding activity (Fig. 4f).

### AC-186 promotes deacetylation of p65 sub-unit of NF-κB in LPS-stimulated BV-2 microglia

Results in Fig. 4g and h indicate that protein levels of acetyl-NF-κB p65 were significantly (*p* < 0.001) elevated in BV-2 cells stimulated with LPS (100 ng/mL), in comparison to unstimulated BV-2 microglial cells. However, AC-186 treatment prior to LPS stimulation resulted in significant (*p* < 0.001) deacetylation of NF-κB p65 with protein levels falling to 42.4%, 34.1%, 28.1% and 20.9% in the presence of 0.625, 1.25, 2.5 and 5 µM of the compound, respectively (Fig. 4g and h).

### LPS-indued repression of SIRT-1 protein expression was reversed by AC-186

**Fig. 5:**
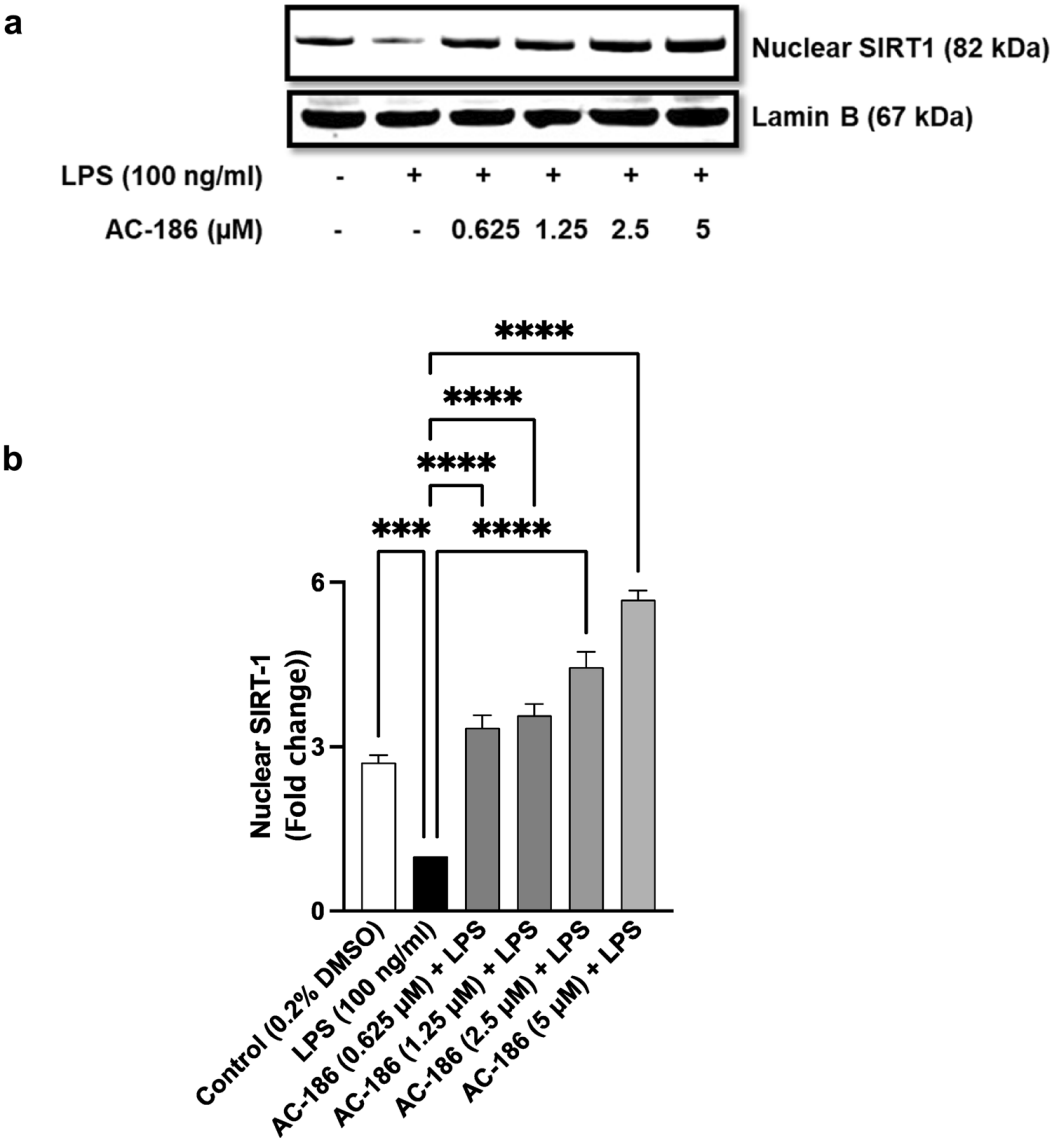
AC-186 increases SIRT-1 protein in BV-2 microglia. Microglia cells were treated with LPS (100 ng/mL) in the absence or presence of indicated concentrations of AC-186 followed by immunoblotting of nuclear extracts for SIRT-1. Lamin B was used as an internal loading control. Data are expressed as the mean ± SEM (n = 3). ****p < 0.0001 versus LPS stimulation alone

SIRT-1 is a class III histone deacetylase (HDAC), which is involved in the deacetylation and regulation of the transcriptional activity of NF-κB p65. Encouraged by results showing that AC-186 prevented LPS-induced acetylation of NF-κB p65, experiments were conducted to evaluate effects of the compound on SIRT-1 protein expression in LPS-activated BV-2 microglia. Results in Fig. 5a and b depict significant (*p* < 0.001) suppression of nuclear SIRT-1 protein levels in cells stimulated with LPS, when compared with unstimulated cells. Significant (*p* < 0.001) reversal of LPS-induced suppression of nuclear SIRT-1 protein was reversed in the presence of AC-186, with ~ 3.3, ~ 3.6, ~ 4.5, and ~ 5.7-fold increase in expression produced by 0.625, 1.25, 2.5 and 5 µM concentrations of the compound, respectively (Fig. 5a and b).

### AC-186 increased oestrogen receptor beta (ERβ) expression and transcriptional activity of oestrogen-response element (ERE) in BV-2 cells

**Fig. 6:**
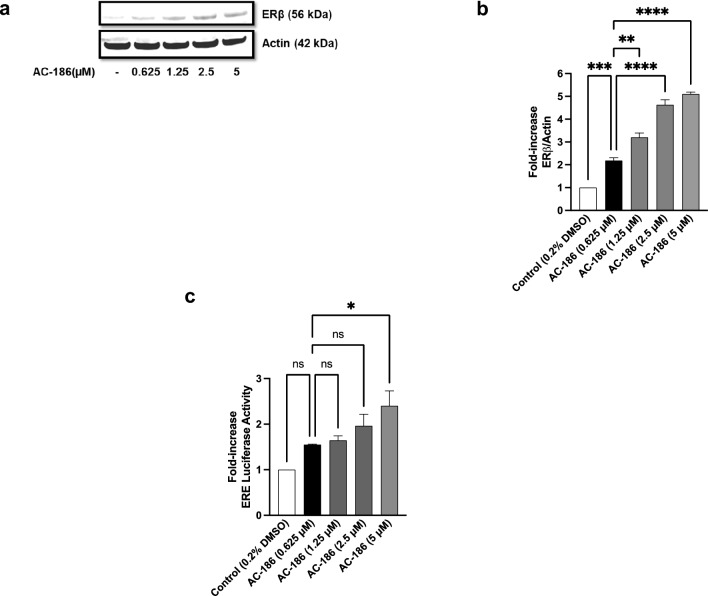
Implications of treating BV-2 microglia with AC-186 on ERβ. **a**, **b** ERβ protein expression was significantly elevated when cells were treated with AC-186. **c** AC-186 (5 μM) increases ER-dependent luciferase activity in BV-2 microglia. Data are expressed as the mean ± SEM (n = 3). ns-not significant at *p* < 0.05, *p < 0.05, ****p < 0.0001 versus control alone

Based on reports indicating that AC-186 is a selective non-steroidal oestrogen receptor β agonist [10], and studies suggesting that oestrogen modulates neuroinflammation by interacting with ERβ [16], experiments were conducted to determine whether AC-186 could affect protein expression of ERβ in BV-2 microglia. Figure 6a and b show that ERβ is expressed in BV-2 microglial cells and treating these cells with 0.625, 1.25, 2.5 and 5 µM of AC-186 resulted in ~ 2.2, ~ 3.2-fold, ~ 4.6-fold and ~ 5.1-fold increase in expression of ERβ protein, respectively when compared to control cells. Interestingly, results of reporter gene assays revealed that significant (*p* < 0.05) increase in ERE transcriptional activity was only produced in the presence of 5 µM of the compound while treatment of BV-2 microglia with lower concentrations of the compound resulted in insignificant (*p* < 0.05) increase (Fig. 6c).

### Anti-inflammatory effects of AC-186 in BV-2 microglia are dependent on ERβ

**Fig. 7:**
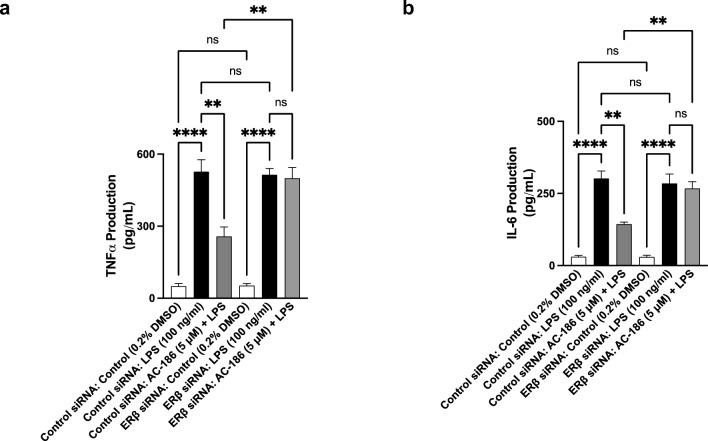
Effects of ERβ knockdown on anti-inflammatory effects of AC-186. Control siRNA- and ERβ siRNA-transfected BV-2 cells were treated with AC-186 (5 μM) prior to stimulation with LPS (100 ng/mL) for 24 h. Culture supernatants were analysed for TNFα **a** and IL-6 **b**. Data are expressed as the mean ± SEM (n = 3). ns-not significant at *p* < 0.05, **p < 0.05, ****p < 0.0001

Studies have provided evidence linking anti-inflammatory action of oestrogen to its interaction with ERβ. Results of anti-inflammatory effects of AC-186, coupled with its reported ERβ agonist activity prompted investigations to determine whether its anti-inflammatory effect was dependent on ERβ. Results in Fig. 7 show that there were significant decreases in both TNFα (Fig. 7a) and IL-6 (Fig. 7b) levels in control siRNA-transfected BV-2 microglia which were treated with AC-186 (5 µM) and stimulated with LPS (100 ng/mL). In ERβ siRNA-transfected cells however, AC-186 (5 µM) failed to reduce TNFα and IL-6 production following stimulation with LPS.

### Incubation of LPS-activated BV-2 microglia with AC-186 prevented neuroinflammation-mediated neuronal damage

**Fig. 8:**
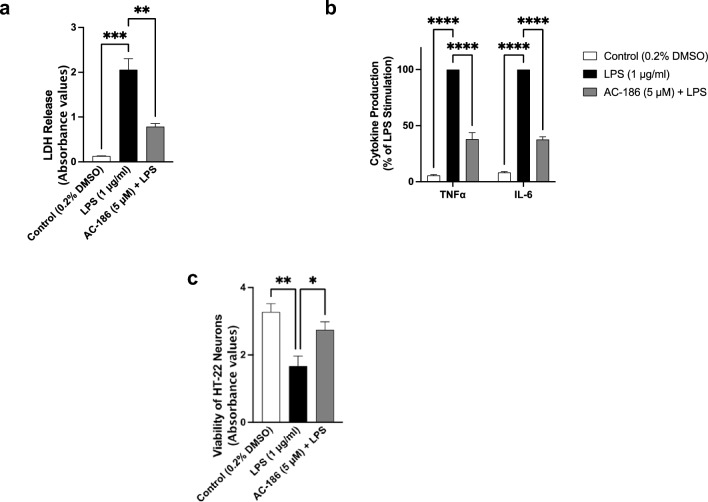
Neuroprotection by AC-186 in microglia-neuron co-culture. BV-2 microglia-HT-22 neurons were co-cultured in a transwell system and treated with AC-186 (5 μM) prior to stimulation with LPS (1 μg/mL) for 48 h. **a**. Neuronal viability was determined using LDH assay. **b**. Reduction in the levels of TNFα and IL-6 were measured using mouse ELISA kits. **c**. Viability of neurons was assessed using MTT assay. Data are expressed as the mean ± SEM (n = 3). **p < 0.01, ***p < 0.001, ****p < 0.0001 versus LPS stimulation alone

Excessive production of pro-inflammatory mediators from the microglia has been proposed as one of the principal mechanisms involved in neuronal damage. Exposing BV-2 microglia co-cultured with HT-22 hippocampal neurons to a high concentration (1 μg/mL) of LPS resulted in significant (*p* < 0.001) increase in LDH release by the neurons when compared with unstimulated cells (Fig. 8a), suggesting neuronal death. Interestingly, supernatants collected from the BV-2 layer showed significant (*p* < 0.0001) increase in levels of both TNFα and IL-6 (Fig. 8b). However, pre-treating the microglial layer with AC-186 (5 μM) resulted in significant (*p* < 0.01) reduction in LDH release by the neurons, while levels of TNFα and IL-6 in microglial culture supernatants were also reduced (Figs. 8a and b). MTT assay to assess viability of HT-22 neurons showed that activation of BV-2 microglia with LPS (1 µg/mL) resulted in a significant (*p* < 0.01) reduction in the number of viable adjacent neurons, when compared to neurons co-cultured with unstimulated cells (Fig. 8c). However, neuronal viability was improved (*p* < 0.05) when BV-2 cells were treated with AC-186 (5 μM) prior to LPS stimulation (Fig. 8c).

## Discussion

Accumulating evidence continues to suggest a crosstalk between oestrogen and inflammatory signalling pathways in a manner that suggests negative modulation of inflammation by oestrogen signalling [17–19]. Also, immune cells such as macrophages, including the microglia are known to express oestrogen receptors, while several reports have suggested that the anti-inflammatory activities of 17β-oestradiol (E2) and selective oestrogen receptor modulators (SERMs) may be linked to modulation of these receptors [16, 20–23]. AC-186 is a selective non-steroidal ERβ agonist which has been previously reported to produce neuroprotective activity in animal models [10–12]. This study was therefore initiated to determine whether inhibition of neuroinflammation could be a contributing factor in the neuroprotective action of this compound.

Excessive release of pro-inflammatory cytokines such as TNFα and IL-6 is one of the principal neuron-damaging factors in activated microglia during neuroinflammation. In fact, elevated levels of TNFα have been linked to CNS disorders such as Alzheimer’s disease [24], Parkinson's disease [25, 26], and multiple sclerosis [27]. In this study, AC-186 reduced neuroinflammation by dampening excessive production of both TNFα and IL-6 in LPS-activated BV-2 microglia, thus suggesting a potential disease-modifying strategy in neurodegenerative disorders.

In addition to the pro-inflammatory cytokines, elevated microglial secretion of iNOS-generated nitric oxide and PGE_2_ have been linked to neurodegeneration [28, 29]. The outcome of this study showed that AC-186 attenuated marked secretions of NO and PGE_2_ as well as the accompanying increased protein expression of both iNOS and COX-2 in LPS-activated BV-2 microglia, thus further confirming anti-inflammatory activity of the compound.

Oestradiol has been widely reported to produce anti-inflammatory action in the microglia [16, 21]. However, there are limitations in the clinical application of this hormone as a neuroprotective strategy because of its potential adverse effects on reproductive organs as well as risks of endometrial and breast cancer. On the other hand, SERMs like tamoxifen and raloxifene have been shown to reduce release of pro-inflammatory mediators from microglia [30–33]. The outcome of this study showing inhibition of neuroinflammation by AC-186 therefore confirms that anti-inflammatory SERMs are worthy of further investigation as potential neuroprotective strategies in neurodegenerative disorders.

Based on the established regulatory roles of NF-κB on the genes encoding pro-inflammatory mediators such as TNFα, IL-6, iNOS and COX-2, as well as reports of crosstalk between this transcription factor and oestrogen receptor, it became necessary to determine whether mechanism(s) of the anti-inflammatory action of AC-186 involved modulating NF-κB signalling in LPS-activated BV-2 microglia. This study showed that the negative modulatory effect of AC-186 on LPS-induced activation of the NF-κB signalling was obvious in its ability to block phosphorylation of both NF-κB p65 sub-unit and its inhibitor protein IκB in the cytoplasm, as well as transcriptional activity in the nucleus.

It has been suggested that the interactions between NF-κB and oestrogen receptors may explain the anti-inflammatory activities of oestradiol and SERMs like AC-186. Studies have shown that transcriptional upregulation of pro-inflammatory cytokines like IL-6 could be blocked through oestrogen receptor-mediated interference with NF-κB binding to the IL-6 promoter [17, 19, 34]. It is proposed that AC-186 may be targeting NF-κB signalling to inhibit neuroinflammation by an indirect agonist activity of ERβ receptors [10–12]. Interestingly, results from this study showed that AC-186 increased both the expression and activity of oestrogen receptors in BV-2 microglia which may further explain the anti-inflammatory of the compound.

One of the critical steps involved in the activation of NF-κB and regulation of gene transcription is histone acetylation of the p65 sub-unit. Therefore, an important strategy in targeting neuroinflammation is to achieve deacetylation of p65. This study established that LPS-induced acetylation of p65 was inhibited by AC-186. Deacetylation achieved by AC-186 was further confirmed in experiments showing that the compound increased the nuclear protein expression of SIRT-1. This is an intriguing outcome as SIRT-1, a member of the sirtuin family, is known to induce deacetylation of the p65 sub-unit at lysine 310. Consequently, AC-186 may be producing deacetylation of NF-κB through mechanisms involving elevation of SIRT-1 expression. This outcome reflects reports on studies with the rat brain, which showed that 17β-oestradiol stimulated SIRT1 to inhibit acetylation of p53 as a mechanism of its neuroprotective activity against ethanol-induced neuronal damage [35], thus confirming a role for SIRT-1-mediated deacetylation in neuroprotection by oestrogenic compounds.

Neuroinflammation-mediated neuronal damage was assessed in a BV-2 microglia-HT-22 neuron co-culture in which increases in the secretion of TNFα and IL-6 by microglia were used as criteria for neuroinflammation. Using a transwell co-culture model, it was observed that AC-186 produced neuroprotective effect against neurodegeneration induced by microglia activation. This outcome further confirms the anti-inflammatory effect of AC-186, while demonstrating that this attribute of the compound may be responsible in part for its neuroprotective effects in rodent models of Parkinson’s and Alzheimer’s diseases [10, 11].

## Conclusions

This study established that AC-186 produces NF-κB-mediated anti-inflammatory activity, which is proposed as a mechanism involved in its reported neuroprotective effects in rodent models of neurodegenerative disorders. Like oestrogen and some SERMs, the anti-inflammatory activity of this compound is possibly linked to its agonist effect on ERβ.

## Supplementary information

Below is the link to the electronic supplementary material.

Supplementary file 1 (DOCX 112 KB)

## Data Availability

No datasets were generated or analysed during the current study.
