# Supplementary figures and images for: Inhibition of neuroinflammation and neuronal damage by the selective non-steroidal ERβ agonist AC-186
Source: Inflamm Res. 2024 Oct 3;73(12):2109–21. doi: 10.1007/s00011-024-01952-y (PMC11632062; doi:10.1007/s00011-024-01952-y)

**Supplementary Data**

**ERβ Knockdown Efficiency**

**
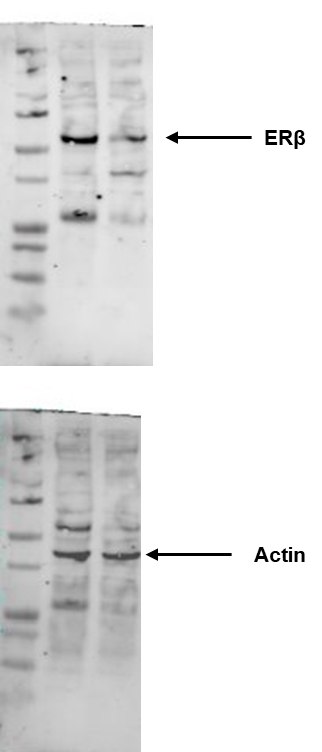
**

Supplement: Supplementary file 1 — (DOCX 112 KB) [file 11_2024_1952_MOESM1_ESM.docx]
